# A Medical Causerie

**Published:** 1915-10-30

**Authors:** 


### A MEDICAL CAUSERIE.

The Exhibition of Fracture Apparatus at the Royal Society of Medicine was a great success and is likely to lead to widespread advantages. Of course there was much interest in the presence of Sir Alfred Keogh and Sir Almroth Wright, and from the remarks made by the former it is evident that ideas relative to the organisation of surgical methods are attracting the authorities of the R.A.M.C. Uniformity of practice and continuity of treatment are engaging phrases, and it is more than probable that much may be done in the directions thus indicated. But medical and surgical treatment cannot be imposed by the rigid rules of a drill-book, and much will always have to be left to the discretion of the individual practitioner, even when he is clothed in khaki. At the same time it is only reasonable that in an elaborate organisation there should be some restriction of the vagaries of private judgment, and no one will quarrel with rules so long as these do not hinder initiative and personal enterprise. The visitors to the exhibition were numerous, and the demonstrations had real teaching value. Sir Almroth Wright's lecture was so attractive that the audience asked for more, and the lecturer was nothing loth. Wounds are now to be arranged in new categories, and the purpose of bandages is less to bind them up than to wash them out. Whatever will our first-aid teachers and their pupils say to it all? Incidentally may be noted the illustration which the exhibition afforded of the value of the Royal Society of Medicine, for no other professional organisation would have initiated and carried through such a scheme.

When professional procedures, with their proverbial delicacies, become invaded by laymen, there is often trouble, and this is particularly the case when questions of advertisement arise. Everyone just now is in sympathy with the British Red Cross propaganda, but there will be some amused smiles over the Society's published announcement that the staff of one of their hospitals includes "the best physicians and surgeons in London." The chief victims are, of course, the members of the staff, who may be anxiously wondering whether this *réclame* may not lead to their appearance before the General Medical Council. The medical officers of medical aid societies have found themselves in trouble as a result of similar proclamations, and the authorities in Oxford Street are reported to be no respecters of persons. Even if these put the telescope to their blind eye, what will the censors of the Royal Colleges say? Possibly the sacred name of charity may cover the crime, but it will hardly condone the error of taste. And even if the secretary of the Society does not have a bad quarter of an hour with the staff he is certain to receive some vigorous protests from their friends.

Like other organisations, the Institute of Hygiene has to adapt its schemes to the circumstances of the hour, and hence it promises for the autumn session courses of lectures dealing with first-aid and invalid cookery and so on. But what will the distinguished medical authorities whose names adorn its programme say to the lecturer who, according to the daily Press, recently announced to "a hundred housewives" that badly cooked meat is just as digestible and nutritious as well-cooked meat? If this doctrine is applied to the special dinners which are, it is understood, one of the features of the Institute's activities in time of peace, the lecturer in question would be well advised to absent himself from the entertainment. Hygiene and dietetics are excellent topics, but their rules are not always strictly observed at medical dinners; if now bad cooking is to be prescribed, even the after-dinner orator may lose his faculty for compliment. The Institute appears rather to cultivate dramatic pronouncements, for some little time ago one of the lady lecturers proposed that women should be recruited for the Army. Possibly Lord Derby may have heard of this, but so far he has made no sign.

It must to many be a rather startling experience to find a public hospital of considerable repute advertising that candidates for one of the most important positions in its organisation must profess a particular religious creed, or at least must be members of one selected ecclesiastical organisation. At first sight there is a mediæval quality about the demand, but the spirit of it is not so deeply buried as liberal minds might imagine. For example, it is only within quite recent years that a similar condition has ceased to be enforced even on the medical teachers in one of the best known of our Metropolitan colleges, where no man was allowed to lecture on the therapeutic virtues of calumba and of quassia unless he was prepared to certify himself as sound on the Thirty-nine Articles. This regulation has now disappeared, but it was in full force less than twenty years ago. There is perhaps more excuse for a college than for a hospital, as the latter offers itself as a public institution and makes its appeal for support to all and sundry. Doubtless the hospital in question is ready to receive contributions even from the heterodox and the dissenter, and possibly some subscribers of this order may have something to say on the official exclusion of candidates dwelling in their special theological camps.

Why do the newspapers in drawing attention to appeals made on behalf of the various war funds repeatedly omit the address to which subscriptions should be sent? Somewhere or other in the advertisement columns there is, no doubt, the official statement, together with the necessary address, but this is not always easily found, and in any case time and trouble must be spent in searching for it. The editorial comment is meant to be helpful, but it would be more helpful if it gave the particulars which would help to secure decision and action. Both the hurried reader and the man who is taking his postprandial budget of news may be prompted by the paragraph, and yet may fail to find the advertisement. Why, then, not make it easy for him to translate his generous impulse into generous action?

